# Bacterial biopolymer (polyhydroxyalkanoate) production from low‐cost sustainable sources

**DOI:** 10.1002/mbo3.755

**Published:** 2018-10-22

**Authors:** Amal A. Aljuraifani, Mahmoud M. Berekaa, Azzah A. Ghazwani

**Affiliations:** ^1^ Biology Department, College of Science Imam Abdulrahman Bin Faisal University Dammam Saudi Arabia; ^2^ Environmental Health Department, College of Public Health Imam Abdulrahman Bin Faisal University Dammam Saudi Arabia

**Keywords:** dates, fluorescence microscopy, low‐cost carbon source, PHA yield

## Abstract

Twenty‐six different bacterial strains were isolated from samples taken from different locations Dammam, Saudi Arabia, for screening of their polyhydroxyalkanoate (PHA) production capability. The initial screening was conducted by staining with Sudan Black B and Nile Red, followed by examination under fluorescence and electron microscopes to characterize PHA granule formation. The PHA‐producing bacterial isolates were identified using 16S rRNA gene analyses; the most potent bacterial strain was identified as *Pseudomonas* sp. strain‐P(16). The PHA production capability of this strain in the presence of different low‐cost carbon sources, such as rice bran, dates, and soy molasses, was analyzed. PHA production in the presence of rice bran, dates, and soy molasses was 90.9%, 82.6%, and 91.6%, respectively.

## 1 INTRODUCTION

Polyhydroxyalkanoates (PHAs) are biodegradable polymers that are produced mainly by bacteria in the form of inclusion bodies and act as storage substances inside vegetative cells (Marang, Loosdrecht, & Kleerebezem, 2018; Santiago, Antonio, Alba, & Bernabe, 2018). As PHAs have properties that are similar to those of synthetic polymers, they are widely used in the production of biopolymers or bioplastics (Burniol‐Figols et al., 2018, Montiel‐Jarillo, Carrera, & Suarez‐Ojeda, 2017). They can be produced from many renewable resources under varied environmental conditions, which reduces the cost of production (Huang et al., 2018; Raza, Abida, & Banat, 2018). The level of PHA granule formation may vary between different producer organisms (Zhang, Shishatskaya, Volova, Ferreira da Silva, & Chen, 2018). These differences stem from the variety in substrates, the nature of polymerization, and the diverse metabolic pathways involved in production. PHA can be synthesized by a diverse group of microbes, which relies solely on the types of carbon sources used (Chen & Jiang, 2018).

The major limitation for PHA production is the cost of production. A number of low‐cost carbon sources have been reported to have been used for PHA production and were found to be cost‐effective (Raza et al., 2018). In addition to the amount of carbon, the carbon/nitrogen (C/N) ratio has also been shown to have a profound effect on cell growth and PHA accumulation (Cui, Shi, & Gong, 2017). High C/N ratios have been reported to promote greater PHA accumulation (Silva et al., 2016). There is great need for carbon sources that are sustainable, inexpensive, and readily available (Koller, Marsalek, deSousa, & Braunegg, 2017; Kourmentza et al., 2017). In addition to the choice of substrates, downstream processing is another crucial factor in making PHA biosynthesis more efficient (Koller, Niebelschutz, & Braunegg, 2013; Madkour, Heinrich, Alghamdi, Shabbaj, & Steinbuchel, 2013). Although there have been several reports on the use of agro‐industrial wastes in the production of PHA, there are some limitations in their use, as most PHA‐derived medical products require high purity to minimize toxicity (Koller, 2018; Peptu & Kowalczuk, 2018). Therefore, the present study was focused on the screening of bacterial strains that could potentially be used for production of PHA in the presence of low‐cost carbon sources, such as rice bran, dates, and soy molasses, that are eco‐friendly, readily available sustainable sources.

## 2 METHODOLOGY

### 2.1 Isolation and screening of bacterial strains

Three different soil samples were collected from different locations (a petrol station, a residence, and a garden) in the city of Dammam in eastern Saudi Arabia and stored in polyethylene bags. Ten grams of each sample was collected in a sterile poly bag, sealed, and transferred to the laboratory or stored at 4°C for later analysis. Approximately 1 g of each sample was dispersed in 99 ml of sterile distilled water and agitated gently for 2 min. Subsequently, the liquid portion of the soil suspension was serially diluted and spread on nutrient agar medium containing peptone (5 g/L), beef (3 g/L), NaCl (5 g/L), and agar (15 g/L). The inoculated plates were incubated at 28–37°C for 24–48 hr. The isolated bacterial colonies were further subcultured to obtain pure cultures of each of the respective bacterial strains. Subsequently, the pure cultures were maintained on nutrient agar slants at 4°C for use in later analyses (Singh, Kumari, Mittal, Yadav, & Aggarwal, 2013).

All bacterial candidates were qualitatively analyzed for PHA accumulation using the viable colony screening technique, which involves the application of Sudan Black B dye. For this purpose, nutrient agar plates containing 1% glucose and an ethanolic solution of 0.3% Sudan Black B dye were inoculated with bacterial isolates and incubated for 24 hr at 37°C. All of the Sudan Black B‐positive isolates were subjected to quantification of their PHA production (Aswini, Kavitha, Revathy, & Babujanarthanam, 2014; Sunitha & Ujwala, 2013). The positive isolates were further subjected to Nile staining for confirmation of PHA accumulation. During this process, bacterial smears were stained with Nile Red A stain for 20 min, cleansed with sterile water and allowed to dry, and then viewed with a fluorescence microscope at a wavelength of 490 nm. PHA‐accumulating bacteria exhibited a bright yellowish‐orange color (Bhuwal, Singh, Aggarwal, Goyal, & Yadav, 2013). Twenty‐six different bacterial strains tested positive during the initial screening procedure; strain 16 was selected for further analysis on the basis of the amount of PHA accumulation, the utilization of carbon sources, and the density of cells.

### 2.2 Optimization of *Pseudomonas* sp. strain‐P (16) for PHA production

*Pseudomonas* sp. strain‐P(16) was optimized for PHA production by testing different inoculum concentrations, incubation periods, and carbon and nitrogen sources. The bacterial isolates were inoculated in PHA production media containing (NH_4_)_2_SO_4_ (2.50 g/L), KH_2_PO_4_ (1.50 g/L), Na_2_HPO_4_ (3.50 g/L), MgSO_4_.7H_2_O (0.20 g/L), glucose (20.00 g/L), agar (20.00 g/L), and 1 ml yeast extract trace element solution (1 mM each of FeSO_4_.4H_2_O, CaCl_2_.2H_2_O, MnSO_4_.4H_2_O, ZnCl_2_) and incubated at 37°C for 24 hr. To determine the effects of strain 16 inoculum concentration on growth and PHA accumulation, inoculum concentrations ranging from 2% to 10% were used for a 24‐hr incubation period at 37°C. For the determination of the effects of the incubation period, strain 16 was cultivated on production medium for 12, 24, 36, 48, 60, and 72 hr at 37°C. To determine the effects of different carbon sources, glucose, fructose, lactose, sucrose, and maltose were used at a concentration of 15 g/L with an incubation time of 72 hr. Similarly, to determine the effects of different nitrogen sources, peptone, casein, (NH_4_)_2_SO_4,_NaNO_3_, and yeast extract were used at a concentration of 2 g/L with an incubation time of 72 hr.

### 2.3 Production of PHA biopolymer using low‐cost carbon sources

Three different low‐cost carbon sources, rice bran, date molasses, and soy molasses, were added to PHA production medium as the sole carbon source at concentrations ranging from 5 to 50 g/L (w/v). Approximately 2% (v/v) was used as an inoculum in all experiments, with the pH maintained at 7.0. The inoculated flasks were incubated at 37ºC for 72 hr. At the end of the incubation period, the cells were harvested by centrifugation at 10,000 rcf for 15 min at 4°C and washed aseptically with sterile, distilled water. The cell pellet was then dispersed in an equal quantity of sodium hypochlorite (5.5% active chlorine) and incubated at 45°C for 2 hours. This extract was centrifuged at 8,000 rcf for 20 min, and the PHA pellet was rinsed once with water and twice with a mixture of ethanol and acetone (2:1). Subsequently, the pellet was dissolved in chloroform and centrifuged briefly at 8,000 rcf to remove the undissolved debris. Finally, the purified PHA was allowed to dry, and the PHA yield was determined by calculation of the dry weight (g/L). For estimation of the dry cell weight, the whole cell mixture was again centrifuged at 10,000 rcf for 10–15 min and the supernatant was discarded. The cell pellet was washed with water to remove the residual media. Finally, the cell pellet was allowed to dry overnight at 60°C until a constant weight (g/L) was obtained. The PHA yield was calculated using the following formula: % PHA = (weight of PHA/dry cell weight) × 100.

### 2.4 Identification and characterization of PHA‐producing bacterial strains

For the molecular identification of PHA‐producing bacteria, the positive strains were identified using 16S rRNA gene analysis. The strains were sent to the Institute for Research and Medical Consultation (IRMC) and identified using PCR. Each PCR contained Top *Taq* polymerase (Qiagen, Germany), 10 mM Top Taq PCR buffer, 10 mm dNTPs, forward and reverse primers (Applied Biosystems, Life Technologies Corporation, USA), and distilled water, to which a loopful of each bacterial colony was added. Each reaction was subjected to an annealing temperature of 56°C for 75 s in a MyCycler^TM^ (Bio‐Rad, USA). All of the amplified products were purified using a PCR Purification Kit (Qiagen, Germany). The BigDye Terminator Cycle Sequencing Kit (Applied Biosystems, Life Technologies Corporation, USA) was used to sequence the purified amplicons, which were electrophoresed on a Genetic Analyzer 3500 (Applied Biosystems, Life Technologies Corporation, USA) using POP 7. The EzTaxon tool was used to determine the similarity of the 16S rRNA sequences obtained from the isolated bacteria with reference sequences from various bacterial species (Chun, Vanessa, & Deborah, 2007). Sequencing Analysis Software, version 5.4 (Applied Biosystems, Life Technologies Corporation, USA), was used to verify the absence of background noise. MAFFT, version 7, was used for the manual verification of sequence similarity (Katoh & Standley, 2013). The most potent PHA‐producing bacterial strain that was subjected to molecular identification using 16S rRNA gene analysis is *Pseudomonas* sp. strain‐P (16).

### 2.5 Fourier transform infrared (FTIR) analysis

The extracted PHA was characterized using Fourier transform infrared spectroscopy. Dried PHA polymer from the bacterial candidate was fixed by combining it with KBr powder to form disks. Spectra were obtained between 400 and 4,000 cm^−1^ using the SHIMADZU–IR AFFINITY‐2‐FTIR spectrophotometer (Shimadzu Corporation, Japan) (Kansiz, Jacobe, & Mc Naughton, 2000).

## 3 RESULTS

### 3.1 Isolation and screening of bacterial strains

**Figure 1:**
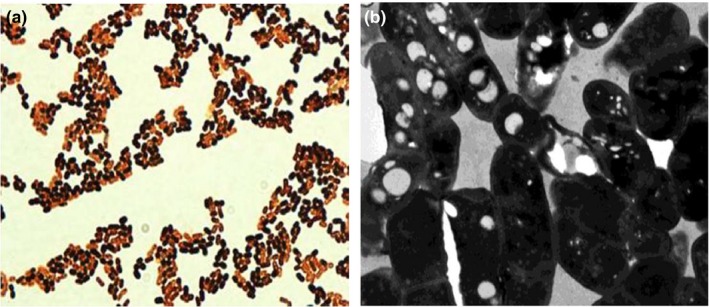
(a) Fluorescence microscopic view exhibiting bright yellowish‐orange color and (b) electron microscopic (EM) view showing polyhydroxyalkanoate granules inside the cells of *Pseudomonas* sp. strain‐P (16)

During the present investigation, a total of 26 bacterial strains were isolated from the Eastern Project in Dammam, Saudi Arabia. Of the 26 isolates, only eight strains were found to be potent PHA producers upon staining with Sudan Black B dye and Nile red stain. Out of these eight potent bacterial strains, *Pseudomonas* sp. strain‐P(16) was found to be the best potential candidate for PHA production on the basis of the size of the spherical granules in the vegetative cells. Most of these granules were found to occupy 50% of the cellular volume and were surrounded by compact membranes (Figure 1). Transmission electron microscopic studies found that optimal production of PHA particles within the bacteria cells occurred in during the 72‐hr‐long trial. The size distribution of the PHA granules ranged from 0.5 to 1.0 μm, with a mean size of 0.5 ± 0.06 μm. The bacterial cells contained large, white inclusion particles within the cytoplasmic fluid, and were stretched and inflated due to the number of particles within them.

### 3.2 Optimization of *Pseudomonas* sp. strain‐P (16) for PHA production

**Figure 2:**
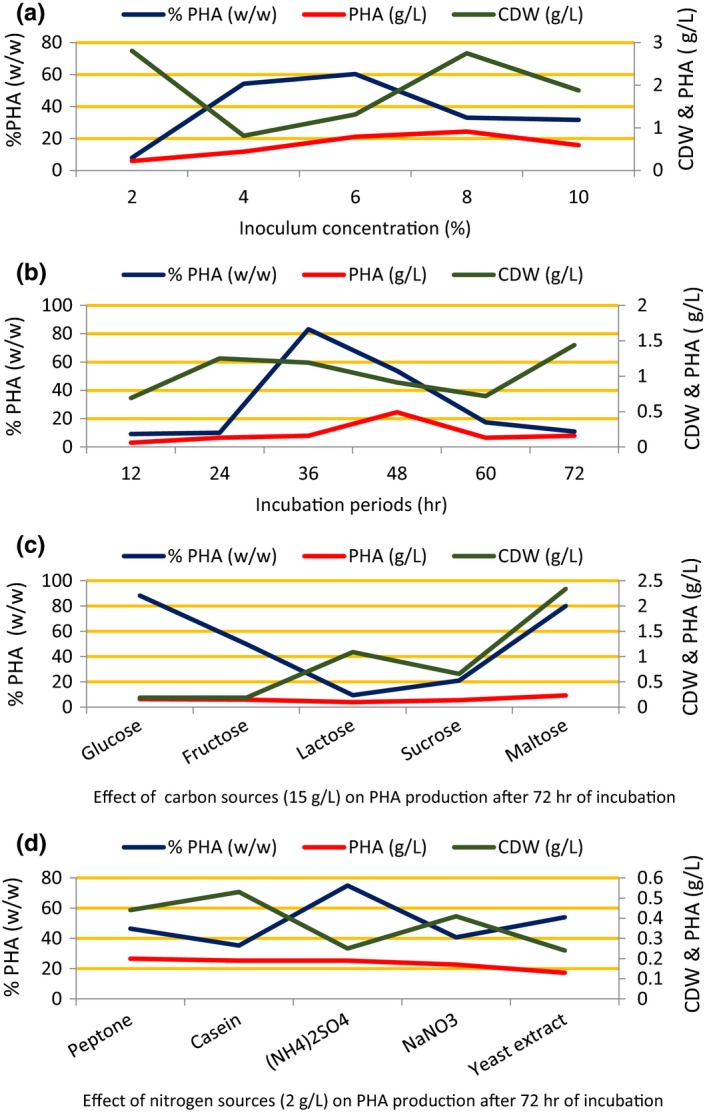
(a–d) Optimization of *Pseudomonas* sp. strain‐P(16) under various growth conditions

To obtain maximum PHA production, the culture conditions for *Pseudomonas* sp. strain‐P(16) were optimized in terms of the inoculum concentration, incubation period, and sources of carbon and nitrogen. Maximum PHA production (55%–62%) was obtained when 4%–6% (v/v) of inoculum was used (Figure 2a). PHA production increased from 10% to 84% with an increase in the incubation period from 12 to 36 hr; beyond 36 hr, there was gradual decline in production (Figure 2b). When the PHA production medium was supplemented with different carbon sources, such as glucose, fructose, lactose, sucrose, and maltose, maximum PHA production (80%–85%) was obtained when either glucose or maltose was used as the sole carbon source (Figure 2c). There was no marked variation in PHA production (36%–54%) when different nitrogen sources were used; an exception was ammonium sulfate, which leads to a maximum production of 79% (Figure 2d).

### 3.3 Production of PHA biopolymer using different low‐cost carbon sources

**Figure 3:**
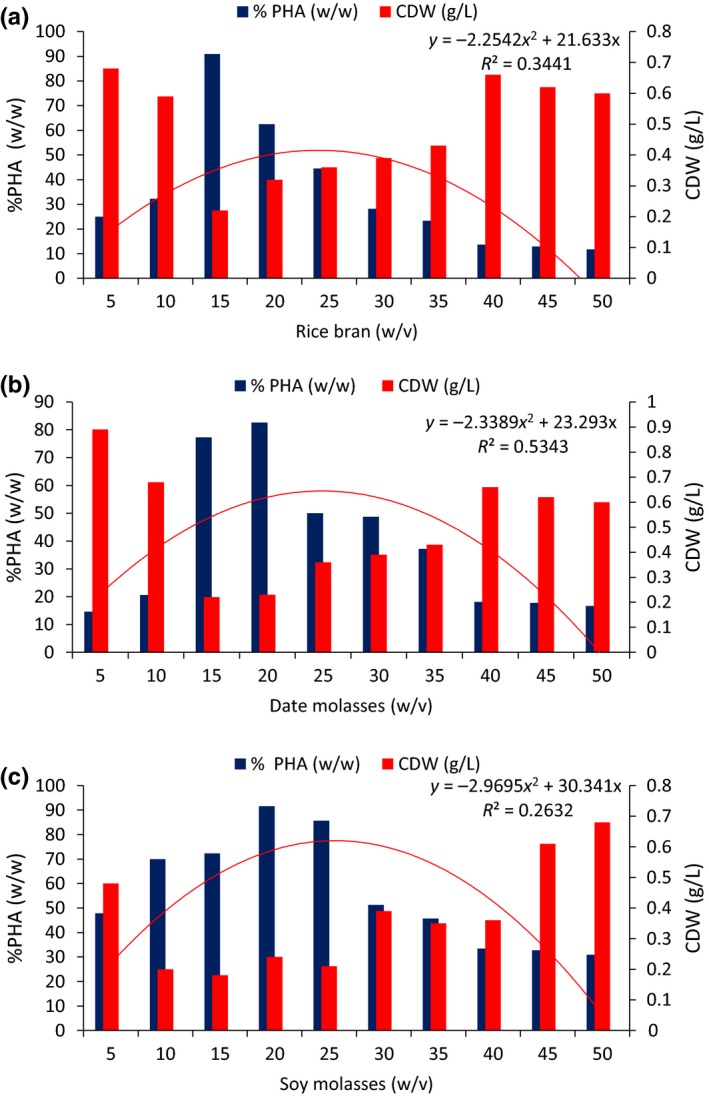
Production of polyhydroxyalkanoate by *Pseudomonas* sp. strain‐P(16) supplemented by (a) rice bran (b) date molasses (c)

In this experiment, PHA production by *Pseudomonas* sp. strain‐P(16) was analyzed after growth for 72 hr in the presence of different low‐cost carbon sources, such as rice bran, date molasses, and soy molasses (Figure 3a–c). The Results showed that when the medium was supplemented with rice bran at a concentration of 15 g/L (w/v), the maximum production of PHA was 90.9% (*R* ^2^ = 0.35), with a CDW of 0.22 g/L. When the medium was supplemented with date molasses, the greatest PHA production (82.6%, *R* ^2^ = 0.53) was obtained at a concentration of 20 g/L, with a CDW of 0.23 g/L. In case of soy molasses, the maximum PHA production was 91.6% (*R* ^2^ = 0.0.26) at a concentration of 20 g/L and a CDW of 0.24 g/L. Based on the above findings, it can be concluded that all three of the carbon sources generate the greatest PHA production at an optimum concentration of 15–20 g/L. The present findings revealed an increase in the CDW that occurred irrespective of the concentration of the carbon sources that was used. It is also pertinent that, with the increase in the PHA production, there was a marked decrease in the CDW values.

### 3.4 Characterization of PHA biopolymer using FTIR analysis

**Figure 4:**
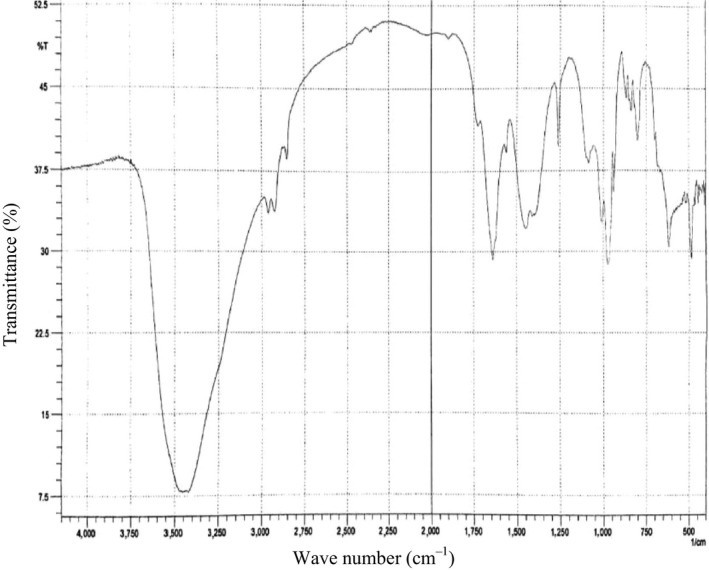
Fourier transform infrared spectra analysis of polyhydroxyalkanoate produced by Pseudomonas sp. strain‐p (16)

Fourier transform infrared spectroscopy was used for the chemical characterization of the extracted polymer. Dried PHA polymer from the bacterial candidate was used to prepare KBr disks. Each chemical complex in the sample has its own unique signature within the absorbance spectrum; hence, the IR spectrum obtained from a sample is representative of its total chemical composition. The discreteness of an independent spectrum that is caused by the chemical structure of every constituent, and the degree to which each contributes to the total spectrum, is directly associated with the concentration of each constituent in the sample. Spectra were recorded between 400 and 4,000 cm^−1^ using a Nicolet 6700 FTIR spectrometer (Nicolet Instrument Corporation, USA). The biopolymer produced by the *Pseudomonas* sp. strain‐P (16) generated two main absorption peaks within the C–H and carbonyl stretching bands that are characteristic of PHA (Figure 4). First, intense hydroxyl stretching at 3456.43 cm^−1^ was observed, which is characteristic of the –OH group. Absorption bands occurring at 2986.44 and 2858.50 cm^−1^ indicated the presence of aliphatic ‐CH_3_ and ‐CH_2_ groups. The absorption bands at 1637.56 and 1261.44 cm^−1^ in the extracted PHA sample corresponded to the C=O and C–O stretching groups and were identical to those produced by PHA that was previously isolated from microbes (Hong, Sun, Tian, Chen, & Huang, 1999).

## 4 DISCUSSION

The current study demonstrated that the selected bacterial strain is capable of producing large amounts PHA by utilizing low‐cost carbon sources. One of the challenges in the production of biopolymers is the associated costs (Chen & Jiang, 2017; Chen & Jiang, 2018). The present findings addressed the possibility of biopolymer production by *Pseudomonas* strain‐P (16) using low‐cost sources, such as rice bran, dates, and soy molasses. These three easily obtainable raw materials can be successfully exploited for bioconversion into high value biopolymer by potent bacterial strains. In the optimization experiment, maximum PHA production was obtained when an inoculum concentration of 4%–6% was used. When different carbon sources were tested, glucose and maltose supported better PHA production. Different nitrogen sources were also tested, among which ammonium sulfate promoted maximum PHA production. In the present study, the optimum concentration for maximum PHA production using rice bran (90.9%) was 15 g/L (w/v); for dates (82.6%) and soy molasses (91.6%), it was 20 g/L (w/v). Huang, Duan, Huang, and Chen (2006) obtained a maximum PHA production of 55.6% using *Haloferax mediterranei* in the presence of extruded rice bran and cornstarch at a ratio of 1:8 g/g. Omar, Rayes, Eqaab, Vob, and Steinbtlchel (2001) obtained a PHB content of 50% (w/w) using *Bacillus megaterium* with date syrup at a concentration of 5% (w/v). Similarly, Ataei, Vasheghani‐Farahani, Shojaosadati, and Tehrani (2008) obtained a maximum PHA production of 71% using date syrup waste. Solaiman, Ashby, Hotchkiss, and Foglia (2006) produced crude PHA at 5%–17% using 2%–5% (w/v) of soy molasses. Similarly, Full, Jung, and Madigan (2006) produced 25.4% PHA using soy molasses at a concentration of 0%–2%. In addition to optimizing PHA production using low‐cost resources, improvement of the overall yield is a major concern (Huang, Chen, Wen, & Lee, 2017). In the present study, the maximum PHA yield recorded was in the range of 73%–92%, which is much greater than that achieved during previous studies (Abid, Raza, & Hussain, 2016; Huang et al., 2006). PHA production by bacterial strains also depends on the carbon source used (Raza et al., 2018). During the optimization experiments, glucose and maltose were found to be suitable carbon sources for *Pseudomonas* strain‐P(16) that improved the PHA yield. Of the three carbon sources used with the P(16) strain, the maximum yield was obtained using soy molasses (92%), followed by rice bran (91%) and date molasses (83%). Thus, the present study revealed that the PHA yield depends on the type of substrate used (Venkateswar‐Reddy et al., 2016).

## 5 CONCLUSION

Though extensive research has been conducted on microbial biopolymer production, its cost compared with that of petroleum‐based plastics is still a major concern in regard to large‐scale production of this natural polymer. Thus, the present study revealed that alternative, low‐cost carbon sources that can be utilized for large‐scale production of microbial biopolymer. The present findings also confirmed that Pseudomonas strain‐P(16) is a potent PHA‐accumulating microbe that may be useful for the economical production of biopolymer. The greatest challenge is the effective recovery of the accumulated PHA and its conversion into the finished product.

## CONFLICT OF INTEREST

The authors declare no conflict of interests.

## AUTHORS CONTRIBUTION

Mrs. Azzah participated in the laboratory work (Isolation and extraction of materials). Dr. Mahmoud contributed to Results and Discussion. Dr. Amal contributed to Results and Discussion and is a corresponding author.

## ETHICS STATEMENT

The research was carried out under the rules of ethics approved by the Imam Abdulrahman Bin Faisal University.

## Data Availability

The data presented in this manuscript are available upon request from the institutional archives of the corresponding author.
